# The Effectiveness of Physiotherapy Interventions for Mobility in Severe Multiple Sclerosis: A Systematic Review and Meta-Analysis

**DOI:** 10.1155/2022/2357785

**Published:** 2022-07-11

**Authors:** Tarub Binshalan, Krishnan Padmakumari Sivaraman Nair, Alisdair McNeill

**Affiliations:** ^1^Department of Neuroscience, The University of Sheffield, 385a Glossop Road, Sheffield, UK S10 2HQ; ^2^College of Applied Medical Sciences, Shaqra University, Saudi Arabia

## Abstract

**Background:**

People with Multiple Sclerosis (pwMS) prioritise gait as the most valuable function to be affected by MS. Physiotherapy plays a key role in managing gait impairment in MS. There is little evidence on the effectiveness of physiotherapy for severe MS.

**Objective:**

To undertake a systematic review and meta-analysis of the literature to identify evidence for the effectiveness of physiotherapy for gait impairment in severe MS*. Methods*. The available literature was systematically searched, using a predetermined protocol, to identify research studies investigating a physiotherapy intervention for mobility in people with severe MS (EDSS ≥ 6.0). Data on mobility related endpoints was extracted. Meta-analysis was performed where a given mobility end point was reported in at least 3 studies.

**Results:**

37 relevant papers were identified, which included 788 pwMS. Seven mobility-related endpoints were meta-analysed. Robot-Assisted Gait Training (RAGT) was found to improve performance on the 6-minute walk test, 10-metre walk test, fatigue severity scale, and Berg Balance Scale. Neither body weight supported training nor conventional walking training significantly improved any mobility-related outcomes.

**Conclusion:**

Physiotherapy interventions are feasible for mobility in severe MS. There is some evidence for the effectiveness of RAGT.

## 1. Introduction

Multiple Sclerosis (MS) is an inflammatory immune-mediated disease characterised by demyelination of axons within the CNS that is frequent in young adults and commonly causes a lifelong disability [1]. 85% of people with MS (pwMS) are concerned about their gait problems [2], and 80% have gait problems 10-15 years after onset of MS [3, 4]. The problems in gait in pwMS are due to muscle weakness, spasticity, fatigue, ataxia, and loss of proprioception [5]. Impairment of mobility reduces physical activity. pwMS are less physically active compared to the general population. Approximately 78% of pwMS are not involved in regular physical activity [6]. pwMS with more advanced disease (Expanded Disability Status Scale (EDSS) of 6 or higher) have less muscular strength, aerobic fitness, and reduced balance compared to those with less severe disease [7]. The disease burden of MS is exacerbated by secondary effects of low levels of physical activity, such as obesity, and increased cardiovascular morbidity.

Exercise intervention in the form of regular aerobic, balance, and strengthening exercise has been shown to be particularly effective to improve mobility for pwMS [8, 9]. They improve MS symptoms, overall fitness, mobility, fatigue, and quality of life (QoL). However, most of the interventions targeted mild to moderate MS patients, and the impact of exercise therapies on those with more severe disabilities is yet limited [7]. This reflects the fact that the PT (physiotherapy) programs commonly used to improve mobility are not feasible for this population. In particular, gait training for severely impaired patients is technically challenging because of their motor weakness and balance abnormalities [10].

While there are many studies on PT interventions for pwMS, there are only a limited number of studies on the effect of PT in people with severe MS. In this review, we sought to answer the question: what are the most beneficial PT interventions to improve walking in people with severe MS (EDSS ≥ 6)?

## 2. Methods

The available literature was systematically searched using a predetermined protocol (https://www.crd.york.ac.uk/PROSPERO/display_record.php?RecordID=204284). The PICO framework was used to structure the design of the systematic review and determine the inclusion and exclusion criteria (Table 1). Studies of interest (including randomised controlled trials (RCT), prospective studies, case-control studies, and cohort studies) investigated the effect of a physiotherapy intervention on mobility endpoints for adults (>18 years old), who are diagnosed with MS with severe mobility disability (reported EDSS score ≥6.0 or narrative description of mobility disability, e.g., use of walking aid). Articles were excluded when written in a language other than English, when more than one intervention (including trials of medication) was used or mobility endpoints were not reported.

### 2.1. Search Strategy

The search strategy and search terms were agreed by 2 researchers (TB and AM) to reflect and address the research question. Titles and abstracts were searched in 3 databases (Scopus, Pedro, and Web of Science) from 2000 till April 2022. The keyword combinations utilised as search terms as follows: “multiple sclerosis” AND “Physical therapy” OR Exercise OR Physiotherapy OR Training OR Rehabilitation OR Neurorehabilitation OR “Virtual reality” OR “Balance training” OR “Robot∗ assisted” OR Exoskeleton OR Aerobic OR “Strength training” OR Resistance OR “Treadmill training” OR “Exercise bike” OR Cycling OR Exergaming OR “Tai Chi” OR “Core stability” OR Yoga OR Pilates OR “Assistive device.” Papers were downloaded into EndNote and duplicates removed. Searches were performed in July 2021; repeated in April 2022. Citation lists of included articles were hand searched and identified studies assessed according to the search strategy.

### 2.2. Study Screening Process

**Figure 1:**
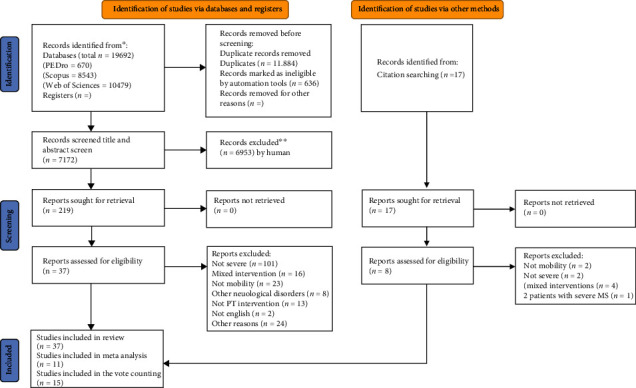
PRISMA flow chart (2020). ^∗^Consider, if feasible to do so, reporting the number of records identified from each database or register searched (rather than the total number across all databases/registers). ^∗∗^If automation tools were used, indicate how many records were excluded by a human and how many were excluded by automation tools. From: Page MJ, McKenzie JE, Bossuyt PM, Boutron I, Hoffmann TC, Mulrow CD, et al. The PRISMA 2020 statement: an updated guideline for reporting systematic reviews. BMJ 2021; 372:n71. doi: 10.1136/bmj.n71. For more information, visit: http://www.prisma-statement.org/

Article screening was guided by the preestablished inclusion/exclusion criteria (Table 1). Two independent reviewers (TB and AM) screened the titles, and 10% of the titles were checked by both reviewers for agreement. Initially, 19 692 papers were identified and 11 884 were removed as duplicates. Title and abstract screening was applied to 7172. The screening process is summarised in Figure 1 (PRISMA chart). Articles, which passed screening, went on to full text evaluation, decisions on inclusion being undertaken in discussion by 2 researchers (TB and AM).

### 2.3. Data Extraction

Table 2 summarizes the participant demographics, intervention characteristics, and pre- and postintervention mobility endpoint data. Studies were grouped according to exercise modalities. All data were collated in Microsoft Excel.

Where studies included a mixed cohort of MS patients with mild, moderate, and severe disease (according to EDSS score) authors were contacted to provide individual data for severe MS participants (Table 3).

### 2.4. Data Synthesis and Meta-Analysis

Cohort demographic descriptive statistics (mean, standard deviation, and range) were calculated using PASW statistics for Windows (IBM). Meta-analysis was completed using the Comprehensive Meta-Analysis software (Biostat, New Jersey). For mobility end-points where results were available for at least 3 studies, meta-analysis was undertaken. Standardised Mean Difference (SMD), 95% confidence intervals and *Z*-score for overall effect were calculated using a random effects model. Heterogeneity was assessed with *I*^2^ statistic. Forest plots were generated to visualise the effect of a given PT intervention on mobility end-points. We assessed the robustness of our results in sensitivity analyses by using fixed-effects models, an alternative statistical metric of mean difference (MD), and by repeating meta-analysis with exclusion of the lowest quality study (largest standard error).

For PT interventions where meta-analysis could not be performed, the intervention was included in a vote counting exercise. The PT intervention was counted as successful if it significantly improved at least one mobility related outcome.

Statistical considerations from the Cochrane Handbook for Systematic Reviews of Interventions were followed to handle missing data. In case of missing standard deviation or standard error, we used the formula SD = SE × √*N*. To obtain the standard deviations in cases where 95% confidence intervals were presented for the small sample size, we followed this formula SD = √*N* × (upper limit − lower limit)/4.128. Where only the median and interquartile ranges are presented. A multiple of 0.75 times the interquartile range or 0.25 times the range was used as a proxy for the standard deviation values, while the median was used as a proxy for the mean.

### 2.5. Quality Assessment

Included studies underwent quality assessment using the Pedro scale (Physiotherapy Evidence database) to assess the methodological quality of the clinical trials (Table 4) [11]. Two reviewers (TB and AM) undertook the quality assessment and resolved differences through discussion.

## 3. Results

### 3.1. Study Selection

A total of 19,692 studies were identified via searches of 3 databases and reference lists (Figure 1). Thirty-seven articles were selected for data extraction (Table 2) [12–48]. In 20 articles, authors were contacted and asked to provide data for severe MS patients from their cohort and only 4 authors responded (Table 3).

### 3.2. Critical Appraisal

Table 2 presents a summary of the 37 included studies. In total, these include 788 MS patients, with 59.6% female and a mean age of 51.88 (standard deviation 3.54). These studies assessed 11 different PT interventions, including robot-assisted-gait training (RAGT) (17 studies), body-weight-supported treadmill training (BWSTT) (5 studies), home-based-exercise (resistance and task specific training) (2 studies), electrical stimulation (2 studies), conventional exercise training (resistance and aerobic exercise) (3 studies), community-based exercise (1 study), total body recumbent stepper training (1study), blood flow restriction (2 study), exergaming (1study), assistive device training (1 study), community exercise (2 study), and ankle robotic training (1 study). In 7 studies, conventional walking training (CWT) was used in a control arm of severe MS patients.

These studies reported more than 15 distinct mobility endpoints, including 6-minute walk test (6MWT) (16 studies), 25-Foot Walk Test (T25FW) (7 studies), Timed Up and Go (TUG) (11 studies), 10-Meter Walk Test (10WMT) (7 studies), 2-minute walk test (2MWT) (5 studies), step length (4 studies), stance phase (%) (4 studies), swing phase (%) (4 studies), total double support phase (2 studies), stride length (2 studies), 20-meter walk test (1 study), five times sit to stand (1study), fast walking speed (1 study), self-selected walking speed (1 study), step length ratio (SLR) (2 studies), step time (1 study), 3- minute walking speed (1 study). Other mobility-related clinical rating scales reported included the Berg Balance Scale (BBS) (4 studies), the Fatigue Severity Scale (FSS) (10 studies), and Multiple Sclerosis Impact Scale (MSIS-29) (6 studies).

From 17 distinct mobility-related endpoints that were reported, only the 6MWT, T25FWT, TUG, 10WMT, BBS, and FSS were described in at least 3 studies of the same PT intervention to enable meta-analysis to be undertaken. We meta-analysed mobility-related endpoints that are related to patients' ambulation, to investigate which PT intervention could alleviate issues that affect mobility in pwMS.

### 3.3. Quality Assessment

Using the Pedro scale, 1 study was rated “excellent” (score 9-10), 12 studies were rated “good” (score 8-6), 15 studies were rated “fair” (score 6-4), and 9 studies were rated “poor” (score<4). The quality assessment is summarised in Table 4.

### 3.4. Meta-Analysis Results

#### 3.4.1. Robot-Assisted Gait Training (RAGT)

**Figure 2:**
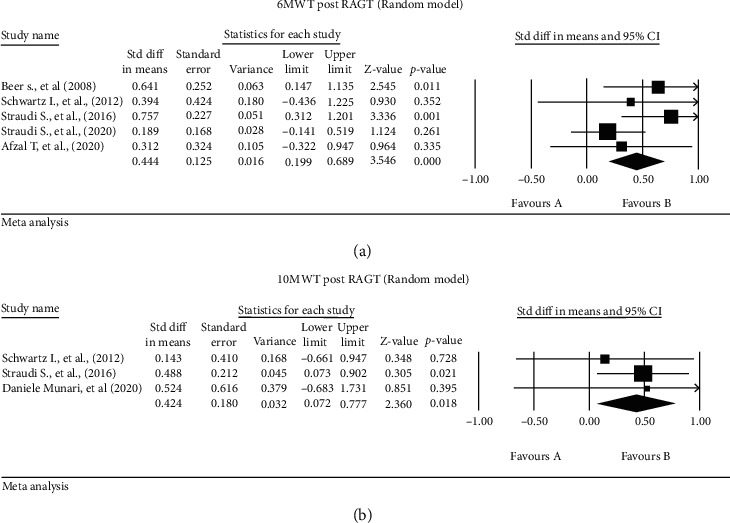
Standardised mean differences in (a) 6MWT and (b) 10MWT post-RAGT.

Five studies [14, 22, 29, 36, 43] reported the effect of RAGT on the 6MWT (*n* = 96 patients), with a significant improvement post-intervention (SMD 0.444, 95% CI [0.199-0.689], *P* ≤ 0.001, *I*^2^ = 19.49%) (Figure 2(a)). The mean increase in 6MWT of all included studies achieved the MCID with a 7% increase in the distance walked except for one study by Afzal et al. [36]. After sensitivity analysis by excluding the lowest quality studies [22, 36], the result remained statistically significant (SMD 0.498, 95% CI [0.124-0.873], *p* = 0.009, *I*^2^ = 58.43%). An alternative analysis using the fixed model and the mean difference showed similar results (MD 9.030, 95% CI [4.944-13.117], *p* ≤ 0.001, *I*^2^ = 38.92%).

**Figure 3:**
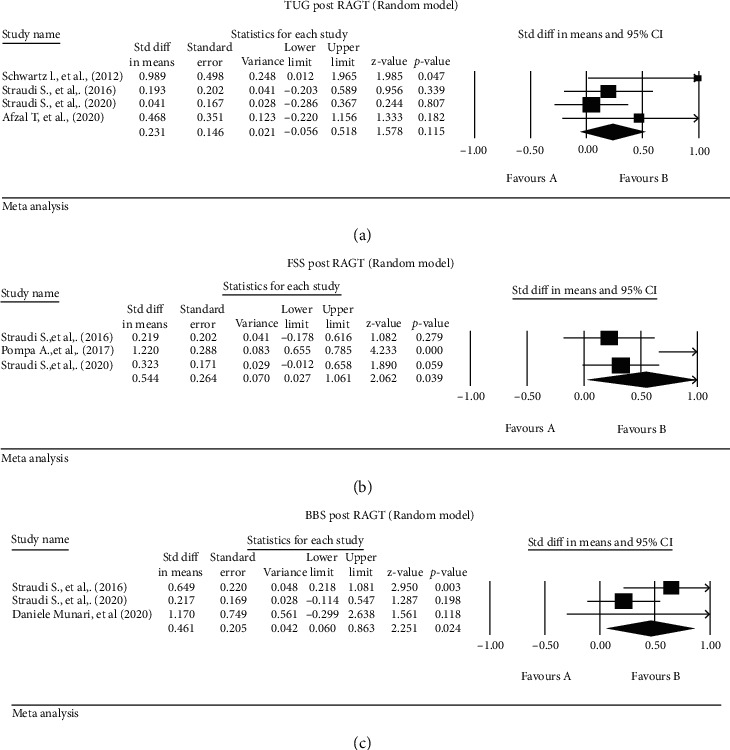
Standardised mean differences in (a) TUG, (b) FSS, and (c) BBS post-RAGT.

Three studies [22, 29, 42] reported the effect of RAGT on the 10WMT (*n* = 34 patients), with a significant effect postintervention (SMD 0.424, 95% CI [0.072-0.777], *p* = 0.018, *s*%) (Figure 2(b)). Four studies [22, 29, 36, 43] described a nonsignificant effect of RAGT on TUG (*n* = 76 patients) (SMD 0.2, 95% CI [-0.056-0.52], *p* = 0.155, *I*^2^ = 24.9%) (Figure 3(a)).

Three studies [29, 31, 43] reported a significant effect of RAGT on the FSS postintervention (*n* = 82 patients) (SMD 0.54, 95% CI [0.027-1.06], *p* = 0.039, *I*^2^ = 77.7%) (Figure 3(b)). Sensitivity analysis by using the fixed model and the mean difference showed also similar results (MD 0.596, 95% CI [0.350-0.843], *p* ≤ 0.001, *I*^2^ = 85.39%).

Three studies [29, 42, 43] reported a significant effect of RAGT on the BBS post-intervention (*n* = 64 patients) (SMD 0.46, 95% CI [0.06-0.863], *p* = 0.024, *I*^2^ = 43%) (Figure 3(c)). Sensitivity analysis by using the fixed model and the mean difference showed also similar results (MD 2.646, 95% CI [1.330-3.962], *p* ≤ 0.001, *I*^2^ = 0%).

#### 3.4.2. Body Weight-Supported Treadmill Training (BWSTT)

**Figure 4:**
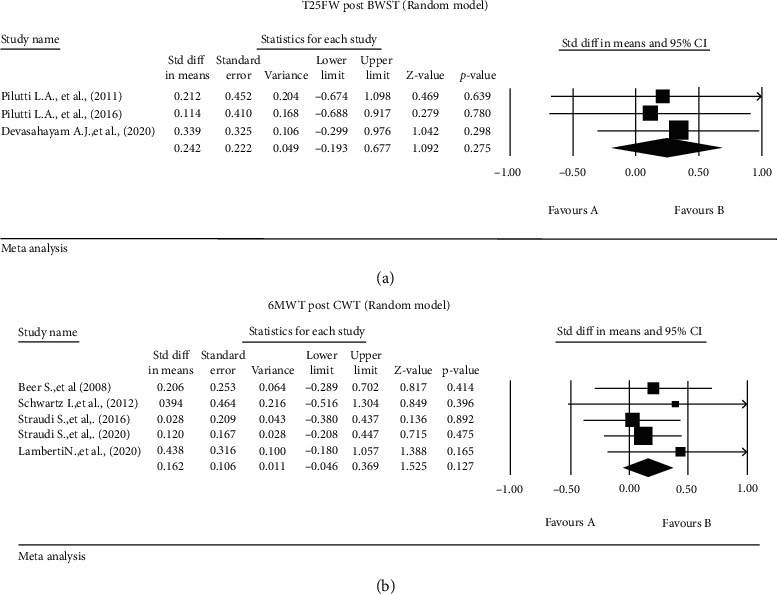
Standardised mean differences: (a) 25-foot walk test after body weight-supported training and (b) 6MWT postconventional walking training.

Three studies [20, 28, 39] described the effect of BWSTT on the T25FW for (*n* = 21 patients), showing no significant effect of intervention (SMD 242, 95% CI [-0.192-0.677], *p* = 0.275, *I*^2^ = 0%) (Figure 4(a)).

#### 3.4.3. Conventional Walking Training (CWT)

Five studies [14, 22, 29, 40, 43] examined the effect of CWT on the 6mwt for (*n* = 91 patients), showing no significant effect (SMD 0.162, 95% CI [-0.046-0.369], *p* = 0.127, *I*^2^ = 0%) (Figure 4(b)).

### 3.5. Vote Counting Results

16 interventions from 15 studies (Table 5) were included in the vote counting (RAGT [41], RAGT+VR [42], CWT [31, 41], BWSTT+antigravity treadmill training [35],BWSTT [13], BWSTT+dual tasks [32], blood flow restriction [38, 40], total body recumbent stepper training [28], home exercise [19], exergaming [27], balance training [27], ADSTEP [33], group physiotherapy [26, 44], 1 : 1 physiotherapy [26], yoga [26], and kickboxing [21]).

Seven interventions from 6 studies were defined as beneficial in improving mobility in severely disabled MS patients (RAGT, CWT, BWSTT+ dual task training, BFR, group physiotherapy, 1 : 1 physiotherapy and Yoga) [26, 31, 32, 40, 41, 44].

The significant findings from the vote counting results can be summarised as follows: A study by Manfredini et al. [41] showed that RAGT and CWT significantly increased the distance in 6MWT. The Rivermead Mobility Index was significantly improved post CWT in severely disabled MS patients [31]. A study by Jonsdottir et al. [32] reported that a treadmill with dual task training significantly improved 2MWT. Blood flow restricted walking study demonstrated a significant improvement in the 6MWT, walking speed, 5 time sit to stand, and FSS [40]. In addition, a study by Williams et al. [44] found that group physiotherapy significantly improved the 10WMT. Hogan et al.'s study [26] with three intervention groups showed that individual physiotherapy significantly improved 6MWT, balance, and fatigue and group physiotherapy significantly improved balance and fatigue, while yoga only improved balance significantly after the intervention.

## 4. Discussion

We report a systematic review and meta-analysis of the evidence for PT interventions to improve walking performance in severely disabled pwMS (defined as EDSS ≥ 6.0). We include 37 studies that investigated a range of PT interventions in 788 pwMS. Forty three percent of studies included only severely affected pwMS (EDSS ≥ 6.0), and 57% had mixed cohorts with mild, moderate, and severely affected pwMS. Overall study quality was variable; with only 1/37, study rated “excellent,” 12/37 included papers rated “good” on the Pedro scale and 9/30 rated poor. Weaknesses of study design included lack of blinding (for participant, therapist and/or assessor), nonconcealment of allocation, missing data, and lack of intention-to-treat analysis.

A major concern limiting the use of PT interventions for mobility in severely disabled pwMS is increasing the barrier to exercise [49], in particular, health and cognitive barriers. As disease progresses, the health and cognitive status of patients are significantly impaired. Moreover, severely disabled PwMS suffer from fatigue, mobility disability, depression, safety concerns, and hesitation to engage in tasks they cannot perform as simply or effectively as they did previously and an inaccessibility to appropriate places. Moreover, they are uncertain of their capacity to engage in physical activity [49]. Therefore, they need more social and physical support to overcome these obstacles compared to other less affected pwMS [49]. However, our systematic review indicates that severely disabled pwMS can utilise a variety of PT interventions. Of note, 27/37 (73%) studies had a dropout rate of less than 15%, suggesting that the majority of people with severe MS can complete PT protocols for mobility.

There was significant heterogeneity in the mobility outcome measures and statistical analyses reported. There were more than 15 mobility-related outcome measures reported in 37 studies, but there was little overlap in the outcome measures used between studies. Because of this, we could only meta-analyse 6 outcomes (Figures 2–4) from 11 studies.

Based on our meta-analysis, RAGT is the PT intervention for which there is most evidence of effectiveness to improve mobility in severe MS. RAGT significantly improved scores on the 6MWT [14, 22, 29, 36, 43], 10WMT [22, 29, 42], FSS [29, 31, 43], and BBS [29, 42, 43]. In addition, one paper from the vote counting exercise demonstrated a significant effect of RAGT on the 6MWT [31]. Improvements in 6MWT scores likely reflect improved aerobic capacity and endurance. Improvements in the 10WMT scores reflect increases in walking speed after RAGT [50].

Fatigue in MS is multifactorial, reflecting both physical and psychological factors [13]. It is likely that improved aerobic capacity and endurance could lead to reduce perceived fatigue (as measured by the FSS) [31]. Improvements in the BBS are likely associated with general improvements in mobility after RAGT. In contrast, there was no significant effect of RAGT on the TUG. This is likely because TUG is a demanding function particularly for severely disabled patients that requires the patient to stand upright from sitting position, then walk (acceleration-deceleration), and turn to return to the starting point [51]. Moreover, RAGT is designed to improve dynamic walking as an independent function; it is not targeting tasks like sit-to-stand or turning [52]. This suggests that RAGT may need to be complemented with additional PT interventions to target activities like getting up from sitting or transfers.

Seven studies with (*n* = 150 patients) reported the use of CWT in severely disabled pwMS. However, meta-analyses of 6MWT did not show any improvement post-CWT. CWT as a stand-alone intervention might not improve the distance walked in 6 minutes in pwMS. CWT bearing has other advantages like reducing osteoporosis, improving balance, improving self-esteem, and better control of spasticity [29, 53]. It also does not require specialist equipment and could be delivered widely in community [29]. We did not look into these aspects. Further research is required to understand the role of CWT in people with advanced MS.

Five studies investigated the role of BWSTT, though no clear evidence of benefit on mobility related outcomes emerged from our meta-analysis. A number of other PT interventions were identified as being utilised for severely disabled pwMS, including kickboxing [21], exergaming [27], and electrical stimulation [16, 17]. Although there are various rehabilitation programs that work empirically for MS patients, there was relatively few studies on the effect of PT in people with severe mobility impairment [54, 55]. Therefore, further studies are needed particularly for this population.

It may however be noted that the RAGT included studies have been administered to patients with EDSS score ranges (3-7.5), and no study has examined the feasibility of RAGT for patient of EDSS higher than 7.5. On the other hand, 4 studies have been applied to patients with EDSS ≤ 8. Two studies used BWSTT [13, 20] and another study used home Exercise of task-specific programme [19], while Pilutti et al.'s study [28] used total body recumbent stepper training for this population. Although not all severely disabled participants were able to complete the intervention protocol or the outcome measures, the overall effect was positive by increasing the mobility related outcomes and decreasing their disability. We cannot discount that there may be significant numbers of unpublished clinical studies that failed to recruit sufficient numbers of severely disabled pwMS (or in which the intervention could not be completed). Such publication bias may lead to an overestimation of the feasibility of certain PT for severely disabled pwMS. Nonetheless, a range of PT interventions are likely to be feasible for severely disabled pwMS.

There are several factors, which might explain the apparent superiority of RAGT compared to other PT interventions for severely disabled pwMS. Appropriate PT intervention programs must be tailored to the patient's abilities with sufficient stimulus to push present competence to produce effect [49]. Therefore, it possible that RAGT is less demanding for severely disabled pwMS, who might not be able to complete other forms of PT effectively. Moreover, progression in RAGT is easily adjusted by increasing the intensity (frequency and duration) of the training session to challenge patients' abilities [5, 41]. There is evidence that personalised RAGT program might more effectively activate motor areas of the brain and have the potential to induce neuroplastic compensatory mechanisms that might benefit gait and mobility [42]. Moreover, RAGT might also target underlying factors in MS pathogenesis. RAGT has been demonstrated to improve blood mitochondrial function biomarkers, blood oxidative stress markers, and resting muscle oxygen consumption in severely disabled pwMS [41].

There are several limitations to our systematic review. We did not systematically search the grey literature to identify unpublished studies of PT interventions for severely disabled pwMS. We attempted to obtain patient level data on severely disabled pwMS from studies that reported cohorts of mixed MS severity. However, only 4 authors provided the requested data. So, data that could not be obtained was excluded from further analysis. The overall quality of our systematic review and meta-analysis is also influenced by the quality of the included studies. A significant number of included studies were rated “poor” on the Pedro scale. Besides, significant heterogeneity in the mobility-related outcomes was reported. These limited our ability to undertake a meta-analysis.

Our systematic review and meta-analysis provides evidence to guide design of future clinical trials for PT interventions in severely disabled pwMS. The strongest evidence of efficacy is for RAGT. Future clinical trials could focus upon further investigating the effectiveness of RAGT in larger cohorts and defining the most effective and feasible treatment protocols: for example, optimal exercise intensity, duration, and frequency of training episodes. Agreement on a consensus package of mobility-related outcome measures across studies would also be beneficial. There is evidence that longer duration walking tests (e.g., 2MWT) and the Multiple Sclerosis Walking Scale (MSWS-12) are the most sensitive to changes in mobility after PT [56]. A recent systematic review also identified that the 6MWT can discriminate between mild, moderate, and severe MS and in theory measure response to PT [50]. Clinical trials of PT interventions for severely disabled pwMS should be considered a priority given that mobility impairment is considered the most disabling feature of MS by pwMS.

## Figures and Tables

**Table 1:** PICO describing inclusion criteria/exclusion criteria.

Study component	Inclusion criteria	Exclusion criteria
Population	(1) Diagnosed with Multiple Sclerosis according to McDonald criteria (2) Adults (>18 years old) (3) Severe mobility disability (reported EDSS score > 6.0 or narrative description of mobility disability E.G. use of walking aid)	(1) Not MS patients (2) Paediatric participant (<18 years old) (3) Mild-moderate mobility disability (EDSS < 6.0)
Intervention	(1) Physical therapy intervention	(1) Study group includes physiotherapy intervention and concomitant drug or other intervention (2) Study group includes more than one type of physiotherapy
Comparism	No intervention or sham	
Outcomes	Paper reports mobility-related endpoints or outcome	Study reports only nonmobility-related outcomes

**Table 2:** Main characteristics of studies included in the review (including both cohorts with only severe MS and mixed cohorts).

Study characteristics			Participant characteristics				Exercise training characteristics				Outcomes
Ref. (quality)	n	Exercise modality	Gender (% F)	EDSS	Disease duration(y) mean ± SD	Age mean ± SD	Duration (weeks)	Frequency (x/week)	Time (min/session)	Intensity	Outcomes postintervention
Robot-Assisted Gait Training (RAGT) (17 studies)											
Androwis, G. et al. [45]	6	RAGT	50%	Ambulation index ≥2	NR	46.5 ± 5.2	4 weeks	2/week	45 min	Gradually ↓ BWS	↑ cognitive function, ↑ thalamocortical resting-state functional connectivity, ↓ TUG
Berriozabalgoitiaet al., [46]	18	RAGT+gait training Ex.	50%	4.5-7.0	12.94_8.11	49.8 ± 7.26	3 months	2/week	40 min	Gradually ↑ time and ↓ BWS	↓ 10WMT, ↑ balance, ↓ fatigue, ↓ TUG
Druzbicki, M et al. [47]	14	RAGT	57%	5-6	NR	48.08 ± 7.6	3 weeks	5/week	45-60 min	Gradually ↓ BWS	↔ balance, ↓ fatigue, ↓ T25-FW^∗^
Sconza, C.,et al. [48]	10	RAGT+general Ex. (cross-over design)	84.2%	3.5-7	NR	(36-74)	5 weeks	5/week	90 min	40% BWS treadmill speed of 1.5 km (↓ gradually)	↑ 6MWT^∗^, ↓ EDSS, ↓ T25FW^∗^, SLR, ↓ spasticity
Afzal et al. [36]	10	RAGT	80%	6.0-7.5	15 ± 7.1	54.3 ± 12.4	3 weeks	5/week	90 min	Gradually ↑ intensity	↔ 6MWT, ↑ T25FW-self-selected^∗^, ↔ T25FW-fast speed, ↓ NVO2 peak^∗^, ↔ TUG
Berchicci et al. [37]	5	RAGT	40%	5.0-7.0	NR	49.0 ± 7.3	6 weeks	2x/5/week	45 min	NR	↑ T25FW^∗^, ↑ 2MWT^∗^, ↑ Tinetti test^∗^, ↑ BBS^∗^, ↓ fatigue ^∗^, ↑ FSS^∗^, ↑ EBI^∗^, ↓ EDSS^∗^
Daniele Munaria et al. [42]	8	RAGT-VR	62.50%	3.0-6.0	17.7 ± 9.62	57 ± 5.83	6 weeks	2/week	40 min	Gradually ↓ BWS	↑2MWT^∗^, ↓10WMT^∗^, ↑mental function^∗^, ↑BBS^∗^, ↓sway area^∗^
Manfredini et al. [41]	23	RAGT	67%	6.0-7.0	13.30 ± 6.55	56 ± 10	6 weeks	2/week	40 min	Gradually ↑ (distance, speed), ↓ guidance force	↑ 6MWT^∗^, improve mitochondrial function biomarker, ↑ rmVO2
Straudi et al. [43]	36	RAGT	67%	6.0-7.0	12 (6-9)	56 ± 11	4 weeks	3/week	120 min	Gradually ↑ (distance, speed), ↓ guidance force	↓ T25FW^∗^, ↑ 6MWT^∗^, ↓ TUG, ↑ PHQ-9, ↓ FSS, ↑balance^∗^, ↑ QoL, ↑ mental health^∗^
McGibbon et al. [34]	35	Home lower exoskeleton (Keeogo) (cross-over design)	58.60%	4-6.5	NR	49.2 ± 10.6	6 weeks	4 weeks at home (2 weeks with Keeogo, 2 weeks without Keeogo)	All the day	NR	6 MWT + Keeogo < without Keeogo, TUG + Keeogo > TUG without Keeogo^∗^, TST + Keeogo > TST without Keeogo; post 2 weeks with Keeogo at home ⟶ ↑ unassisted 6MWT distance^∗^, ↑ unassisted stair climbing performance^∗^
Pompa et al. [31]	25	RAGT	47.60%	6.0–7.5	17.05 ± 9.12	47.00 ± 11.17	4 weeks	3/week	40	40-50% BWS (↓ gradually)	↑2MWT^∗^, ↑FAC^∗^, ↓EDSS^∗^, ↓FSS^∗∗^, ↑RMI^∗∗^, ↑mBI^∗∗^, ↓VAS^∗^
Straudi et al. [29]	30	RAGT	62.90%	6.0-7.0	13.30 ± 6.55	52.26 ± 11.11	6 weeks	2/week	60 min (30: walking)	100% guidance +50% BWS (↓ gradually)	↑6MWT^∗^, ↓10WMT, ↑BBS^∗^, ↓PHQ − 9^∗^, ↑QoL^∗^, ↓FSS
Straudi et al. [24]	9	RAGT	50%	4.5–6.5	17.1 ± 12.0	49.6 ± 12.0	6 weeks	2/week	60 min (30 min/walking)	Gradually (↑ distance, speed), (↓ guidance force)	Improvements in spatiotemporal parameters (↑gait speed^∗^, ↑cadence^∗^, ↓double support^∗^, ↓step length^∗^ and step time^∗^), ↑6MWT^∗^
Claude Vaney et al. [23]	26	RAGT	NR	3.0-6.5	NR	58.23 (9.42)	3 weeks	3/week	(30 min/walking)	50% BWS (↓gradually), ↑speed to normal gait speed	↑ QoL∗, ↑ 3-minute walking speed^∗^, ↓fatigue^∗^, ↑balance^∗^, ↓spasticity^∗^, ↓activity level, ↔10WMT, ↔RMI, ↔pain level
Schwartz et al. [22]	15	RAGT	57%	5.5–7	11.3 ± 6.7	46.8 ± 12.0	4 weeks	2-3/week	45 min (30 min/walking)	40% BWS (↓ gradually)	↑6MWT, ↓10WMT, ↓TUG^∗^, ↑BBS^∗^, ↓EDSS^∗^, ↑FIM^∗∗^
Beer et al. [14]	19	RAGT	63.20%	6.0–7.5	15.0 ± 8.0	49.7 ± 11.0	3 weeks	5/week	30 min	40–80% BWS, gradually ↑ (distance, speed), ↓ BWS	↑20 m walking velocity^∗^, ↑6MWT^∗^, ↑knee extensor strength^∗^, ↑EBI^∗^
Lo et al. [15]	13	RAGT+BWST (cross-over design)	48% F	3.0 -7.0	NR	49.8 ± 11.1	6 weeks (3 weeks/phase)	2/week	40 min	30%-40% BWS (↓ gradually), ↑ speed	↓T25FW^∗∗^, ↑6MWT^∗^, ↓DST^∗∗^, ↓EDSS^∗∗^, SLR
Body weight-supported treadmill training (BWSTT) (5 studies)											
Devasahayam et al. [39]	10	BWSTT+ cooling room (16°C)	90%	6.0-7.0 (sensitive to heat)	17.6 ± 10.17	53.2 ± 15.6	10 weeks	3/week	40 min	Gradually increased to vigorous intensity (40–65% HRR)	↑fast walking speed^∗^, ↑self − selected walking speed, ↓stance phase (%), ↓swing phase (%), ↓total double support phase, ↓T25FW^∗^, ↓mFIS^∗^, ↓FSS, ↑QoL, ↑aerobic fitness, ↓fatigue^∗^
Willingham et al. [35]	6	BWSTT+ antigravity treadmill training	50%	6.0-6.5	NR	50 ± 4.9	8 weeks	2/week	20 min	35%-70% BWS, speed (0.2–2.5 mph) < RPE of 8.0	↑muscle oxidative capacity^∗^, ↑muscle endurance^∗^, ↑2MWT
Jonsdottir et al. [32]	26	BWSTT if needed+dual task training	44.70%	3.5–7	16.3 ± 7.1	51.4 ± 10.7	4 weeks	5/week	30 min	↑treadmill speed + slope = 14–16 RPE	↑2MWT^∗∗^, ↓10WMT, ↓TUG^∗^, ↑DGI^∗^
Pilutti et al. [20]	6	BWSTT	66%	5.5–8.0	11.5 ± 6.60	48.2 ± 9.30	12 weeks	3/week	30 min	Gradually ↑ speed, ↓ BWS	↓ T25-FW, ↓ fatigue, ↑ QoL, ↓ EDSS
Giesser et al. [13]	4	BWSTT	75%	7.0-8.0	20 ± 5	47 ± 5.3	20 weeks	2/week	60 min	100% BWS (↓ gradually), ↑ speed to normal gait speed	↓ 10WMT, ↑ 6MWT, ↑ balance, ↑ QoL, ↓ spasticity, ↑ muscle strength (not all patients were able to complete the 10WMT, 6MWT)
Total-body recumbent stepper training (TBRST) (1 study)											
Pilutti et al. [28]	5	TBRST	40%	6.0-8.0	15.2 ± 8.9	58.8 (3.0)	12 weeks	3/week	30 min	Gradually ↑ according to participant ability	↓ fatigue, ↑ QoL, ↔ T25FW
Home-based exercise (2 studies)											
de Bolt et al. [12]	19	Home based resistance Ex.	78.95%	1.0 – 6.5.	15 ± 12.23	51.6 ± 7.26	8 weeks	3/week	50 min	Resistance by 0.5% of body weight, ↑ (.05%–1.5%) every 2 weeks	↓ TUG, ↑ leg extensor power^∗^, ↑balance,
Miller et al. [19]	15	Home Ex. of task-specific programme	73.30%	6.5–8	13 ± 9.1	56.3 ± 9.0	8 weeks	2/week	60 min	NR	↑ MSIS-29, ↓ 10WMT, ↑ muscle strength, ↓ timed sit-stand, ↓ EDSS, ↓ FIM
Electrical stimulation (2 studies)											
JE Esnouf et al. [17]	32	FES (ODFS)	61.50%	4.0-6.5	12.5	53	18 weeks	Daily mobility	All the day	NR	↓ effort for walking, ↓ tripping, ↑ confidence while walking, ↑ walking distance
CL Barrett et al. [16]	25	FES (ODFS)	75%	4.0-6.5	13.6 ± 8.3	52.1 ± 6.7	18 weeks	Daily mobility	All the day	Gradually to be worn all the day	↑ walking speed, ↑ walking distance, ↔ physiological cost index
Blood flow-restriction (2 study)											
Chotiyarnwong, C et al. [38]	39	Remote ischaemic preconditioning (RIPC)	44.7%	1.0-7.0	10 ± 10.6	47.6 ± 11.3	1 session	Blood pressure cuff inflated to (30 mm Hg) above resting systolic pressure	Inflation for 5 min followed by deflation for 5 min/3	NR	↑6MWT^∗^, ↑walking speed^∗^, ↓Borg RPE test
Lamberti et al. [40]	12	Blood flow restricted slow walking (BFR-W)	46%	6.1 ± 0.2	14 ± 9	54 ± 11	6 weeks	2/week	60 min	↑ speed by 3 steps/min, BFR stable	↑walking speed^∗^, ↓perceived exertion^∗^, ↑6MWT^∗^, ↓MSIS − 29 (psychological)^∗^, ↓MSIS − 29 (motor), ↓MFIS^∗^, ↓5STS time^∗^
Conventional exercise training (3 studies)											
Resistance training											
S Briken et al. [25]	12	Arm Ergometry	50%	4.0-6.0	17.1 ± 7.2	49.1 ± 8.5	8-10 weeks	2-3/week	15-45 min	Gradually increase	↑6MWT^∗^, ↓fatigue^∗^, ↓depression^∗^
	12	Rowing	36.60%	4.0-6.0	14.1 ± 6.1	50.9 ± 9.2	8-10 weeks	2-3/week	15-45 min		↔ 6MWT
	12	Bicycle ergometry	54.50%	4.0-6.0	13.3 ± 5.4	48.8 ± 6.8	8-10 weeks	2-3/week	15-45 min		↑6MWT^∗^, ↑VO2 peak^∗^, ↓depression^∗^
Hayes et al. [18]	11	Lower limb resistance ex	55.50%	3.5-6.5	11.9 ± 7.3	48.0 (11.9)	12 weeks	3/week	45-60 min	Gradually increase	↑ lower limb strength, ↔ TUG, ↔ 10WMT, ↔ 6MWT, ↑ balance, ↔ fatigue
Aerobic exercise											
Jackson et al. [21]	15	Kick boxing	81.80%	1.0-6.0	12.09 ± 5.5	52.27 ± 8.8	5 weeks	3/week		Gradually increased ≤75% HRR or ≤5 RPE	↑gait speed^∗^, ↓TUG^∗^, ↑balance, ↑Mini − BESTest^∗^
Exergaming (1 study)											
Robinson et al. [27]	20	(Exergaming) Nintendo Wii Fit	70%	6.00	NR	52.6 ± 6.1	4 weeks	2/week	40-60 min	↑ difficulty	↓ postural sway, ↑ balance, ↑ step length, ↑ stride length, ↓ MSWS-12
	18	Balance training	63%	6.00	NR	53.9 ± 6.5	4 weeks	2/week	40-60 min	↑ difficulty	↓ postural sway, ↑ balance, ↑ step length, ↑ stride length, ↓ MSWS-12
Assistive device selection, training and education program (ADSTEP) (1 study)											
Martini et al. [33]	20	ADSTEP	14%	6.0 ± 0 (history of fall)	NR	56.0 ± 9	6 weeks	1/week	40 min	Aid selection, fitting, task-oriented gait training	↓falling^∗^, ↓time spent setting^∗^, ↔TUG, ↔T25FW, ↔2MWT, ↔FSS, ↓MSWS − 12, ↓MSIS − 29, ↑walking aid satisfaction
Community exercise (2 study)											
KL Williams et al. [44]	26	Community group exercise	65.4%	0-5 disease step rating scale	12.4(10.2)	52.7(11.9)	8 weeks	2/week	60 min	↑intensity	↑ 6MWT, ↑ 10WMT, ↑ balance
Hogan et al. [26]	66	Group physiotherapy	62.50%	3–4 on the mobility section of (GNDS)	18(9)	57 (10)	10 weeks	1/week	60 min	Increase the set of (12 repetitions)	↑6MWT, ↑balance^∗∗^, ↑QOL^∗^, ↓MSIS − 29v2 physical component^∗^, ↓MFIS^∗^
	45	1:1 physio-therapy	57%		13(8)	52 (11)	10 weeks	1/week	60 min	Increase the set of 12 repetitions	↑balance^∗∗^, ↑QOL^∗^, ↓MSIS − 29v2 physical component^∗^, ↓MSIS − 29v2 psychological component^∗^, ↓MFIS^∗^, ↑6MWT^∗^
	16	Yoga	61.50%		15(8)	58 (8)	10 weeks	1/week	60 min	NR	↑balance^∗∗^, ↑QOL, ↓MSIS − 29v2 physical component, ↓MSIS − 29v2 psychological component, ↓MFIS, ↑6MWT
Ankle robotic training (1 study)											
Lee Y et al. [30]	7	Ankle robotic training for impaired leg	83.30%	5.2 ± 2.5	16.0 ± 6.5	55.3 ± 11.2	6 weeks	3/week	45 min	NR	↑ROM^∗^, ↑balance^∗^, ↑walking performance, ↑6MWT^∗^, ↓10WMT^∗^

2MWT: 2 minutes walking test; 5TST: 5-time sit to stand; 6MWT: 6 min walk test; 10WMT: Ten-Meter Walking Test; ADSTEP: Assistive Device Selection Training and Education Program; BBS: Berg Balance Scale; BFR-W: Blood flow-restricted slow walking; BWS: body weight support; BWST: body weight supported training; DGI: dynamic gait index; DST: double support time; EBI: Extended Barthel Index; EDSS: Expanded Disability Status Scale; FAC: Functional Ambulation Category; FSS: Fatigue Severity score; FES: functional electrical stimulation; FSST: Four Square Step Test; FIM: Functional Independence Measure; GNDS: The Guy's Neurological Disability Scale; MAS: Modified Ashworth Scale; mBI: modified Barthel Index; mFIS: modified Fatigue Impact Scale; Mini-BESTest: mini Balance Evaluation System Test: MSIS-29: multiple sclerosis impact scale; MSWS: Multiple Sclerosis Walking Scale questionnaire; ODFS: Odstock dropped foot stimulator; PHQ: patient health questionnaire; QoL: quality of life; RAGT: Robot-Assisted Gait Training; RAGT-VR: Robot-Assisted Gait Training combined with Virtual Reality; rmVO2: resting muscle oxygen consumption; RMI: Rivermead Mobility Index; ROM: range of motion; RPE: rating of perceived exertion; SLR: step length ratio; TBRST: Total-Body Recumbent Stepper Training; T25FW: timed 25-foot walk test; TST: timed stair test; TUG: Timed Up and Go; VAS: Visual Analogue Scale.; VO2peak: peak oxygen consumption; NR: not reported. Disease duration in years presented in mean ± SD, otherwise Mean (range). Abbreviations are presented in an alphabetical order. ^∗^Statistically significant at *p* ≤ 0.05 or ^∗∗^*p* ≤ 0.001.

**Table 3:** List of authors who we contacted for data and who responded.

No.	Authors	Responded
1.	Androwis, G. J., et al. [45]	No
2.	Berriozabalgoitia, et al. [46]	No
3.	Druzbicki, M., et al. [47]	No
4.	Sconza, C., et al. [48]	No
5.	Chotiyarnwong, C., et al. [38]	Yes
6.	Berchicci et al. [37]	No
7.	CL Barrett et al. [16]	No
8.	Claude et al. [23]	No
9.	Daniele et al. [42]	Yes
10.	De Bolt et al. [12]	No
11.	Hayes et al. [18]	No
12.	Jackson et al. [21]	Yes
13	JE Esnouf et al. [17]	No
14.	Jonsdottir et al. [32]	Yes
15.	Lee et al. [30]	No
16.	Lo et al. [15]	No
17.	McGibbon et al. [34]	No
18.	S Briken et al. [25]	No
19.	Straudi et al. [24]	No
20.	Berchicci et al. [37]	No

**Table 4:** Pedro scores for included studies (total score out of 10).

No.	Study	Score (out of 10)	Eligibility criteria (external validity)	Random allocation	Concealed allocation	Group similar in baseline	Participant blinding	Therapist blinding	Assessor blinding	<15% dropout	Intention-to-treat	Between-group difference	Point estimate and variability
	Afzal, et al. [36]	2	Y	N	N	N	N	N	N	Y	N	N	Y
	Androwis, G. J., et al. [45]	5	Y	Y	N	N	N	N	Y	Y	N	Y	Y
	Beer et al. [14]	5	Y	Y	N	Y	N	N	N	N	N	Y	Y
	Berchicci et al. [37]	4	Y	Y	N	Y	N	N	N	N	N	Y	Y
	Berriozabalgoitia et al. [46]	5	Y	Y	Y	N	N	N	N	Y	N	Y	Y
	Chotiyarnwong, C et al. [38]	8	Y	Y	N	N	Y	Y	Y	Y	Y	Y	Y
	CL Barrett et al. [16]	5	Y	Y	Y	N	N	N	N	Y	N	Y	Y
	Claude et al. [23]	4	Y	Y	Y	N	N	N	N	N	N	Y	Y
	Daniele et al. [42]	7	Y	Y	Y	Y	N	N	Y	Y	N	Y	Y
	De Bolt et al. [12]	5	Y	Y	N	Y	N	N	N	Y	N	Y	Y
	Devasahayam et al. [39]	1	Y	N	N	N	N	N	N	N	N	N	Y
	Druzbicki, M., et al. [47]	1	Y	N	N	N	N	N	N	N	N	N	Y
	Giesser et al. [13]	2	Y	N	N	N	N	N	N	Y	N	N	Y
	Hayes et al. [18]	5	Y	Y	N	Y	N	N	N	Y	N	Y	y
	Hogan et al. [26]	4	Y	Y	N	N	N	N	Y	N	N	Y	Y
	Jackson et al. [21]	2	Y	N	N	N	N	N	N	Y	N	N	Y
	JE Esnouf et al. [17]	5	Y	Y	N	Y	N	N	N	Y	N	Y	Y
	Jonsdottir et al. [32]	8	Y	Y	Y	Y	N	N	Y	Y	Y	Y	Y
	KL Williams, et al. [44]	7	Y	Y	Y	N	N	N	Y	Y	Y	Y	Y
	Lamberti et al. [40]	8	Y	Y	Y	Y	N	N	Y	Y	Y	Y	Y
	Lee et al. [30]	2	Y	N	N	N	N	N	N	Y	N	N	Y
	Lo et al. [15]	5	Y	Y	N	N	N	N	N	Y	Y	Y	Y
	Manfredini et al. [41]	4	Y	Y	N	N	N	N	Y	N	N	Y	Y
	Martini et al. [33]	7	Y	Y	N	Y	N	N	Y	Y	Y	Y	Y
	McGibbon et al. [34]	6	Y	Y	N	Y	N	N	N	Y	Y	Y	Y
	Miller et al. [19]	6	y	Y	N	Y	N	N	Y	Y	N	Y	Y
	Pilutt et al. [20]	2	Y	N	N	N	N	N	N	Y	N	N	Y
	Pilutti et al. [28]	4	Y	Y	N	N	N	N	N	Y	N	Y	Y
	Pompa et al. [31]	8	Y	Y	Y	Y	N	N	Y	Y	Y	Y	Y
	Robinson et al. [27]	5	Y	Y	N	Y	N	N	N	N	Y	Y	Y
	S Briken et al. [25]	6	Y	Y	Y	N	N	N	N	y	Y	Y	Y
	Schwartz et al. [22]	5	Y	Y	N	Y	N	N	Y	N	N	Y	Y
	Sconza, C., et al. [48]	9	Y	Y	Y	Y	N	Y	Y	Y	Y	Y	Y
	Straudi et al. [29]	7	Y	Y	Y	Y	N	N	Y	N	Y	Y	Y
	Straudi et al. [43]	8	Y	Y	Y	Y	Y	N	N	Y	Y	Y	Y
	Straudi et al. [24]	3	Y	Y	N	N	N	N	N	Y	N	Y	Y
	Willingham et al. [35]	1	Y	N	N	N	N	N	N	N	N	N	Y

**Table 5:** Vote counting of studies for severe MS patients, not included in the meta-analysis.

Study characteristics			Intervention			Outcomes	Vote counting
Studies	*N*	PT intervention	Disability scale	Duration (weeks)	Frequency (X/week)	Outcomes post intervention	Significant mobility-related outcomes
Williams et al. [44]	26	Community group exercise	3-5 disease step rating scale	8 weeks	2/week	↑6MWT, ↑10WMT^∗^, ↑balance	↑10WMT^∗^
Chotiyarnwong et al. [38]	10	BFR	6.0-7.0	1 session	1 day	↑ 6MWT, ↑ waking speed, ↓ perceived exertion	None
Daniele Munaria et al. [42]	5	RAGT+VR	EDSS ≥ 6	6 weeks	2/week	↑ 2MWT, ↓ 10 MW, ↑ BBS, ↑ cognitive function, ↓ double support time, ↓ sway area	None
Manfredini et al. [41]	23	RAGT	6.0-7.0	6 weeks	2/week	↑ 6MWT^∗^, improve mitochondrial function biomarker, ↑ rmVO2^∗^	↑6MWT^∗^
	23	CWT	6.0-7.0		2/week	↑6MWT^∗^, improve mitochondrial function biomarker, ↑rmVO2	↑6MWT^∗^
Lamberti et al. [40]	12	BFR-W	6.1 ± 0.2	6 weeks	2/week	↑6MWT^∗^, ↓5STS time^∗^, ↓mFIS^∗^, ↓MSIS − 29 (psychological)^∗^, ↓MSIS − 29 (motor), ↑walking speed^∗^, ↓perceived exertion^∗^	↑walking speed^∗^, ↑6MWT^∗^, ↓5STS time^∗^, ↓mFIS^∗^
Willingham et al. [35]	6	BWST+antigravity treadmill training	6.0-6.5	8 weeks	2/week	↑2MWT, ↑muscle oxidative capacity^∗^, ↑muscle endurance^∗^	None
Jonsdottir et al. [32]	8	BWST if needed dual task training	EDSS ≥ 6	4 weeks	5/week	↑ 2MWT^∗^,↓ 10WMT, ↑ balance, ↑ DGI,↓ TUG	↑2MWT^∗^
	2	Resistance ex.		4 weeks	5/week	↑ 2MWT, ↓ 10WMT, ↑ balance, ↑ DGI, ↓ TUG	None
Martini et al. [33]	20	ADSTEP	6.0 ± 0 (history of fall)	6 weeks	1/week	↔2 MW, ↓falling^∗^, ↔FSST, ↓MSIS − 29, ↓MSWS − 12, ↔T25W, ↓time spent setting^∗^, ↔TUG, ↑walking aid satisfaction	None
Pompa et al. [31]	25	CWT	6.0–7.5	4 weeks	3/week	↑2MWT, ↓EDSS, ↑FAC ↓ FSS, ↑mBI^∗∗^, ↓↑RMI^∗∗^, ↓VAS	RMI ^∗∗^
Pilutti et al. [28]	5	TBRST	6.0-8.0	12 weeks	3/week	↓ fatigue, ↑ QoL, ↔ T25FW	None
Robinson et al. [27]	20	(Exergaming) Nintendo Wii fit	6.00	4 weeks	2/week	↑ balance, ↓ MSWS-12, ↓ postural sway, ↑ step length, ↑ stride length	None
	18	Balance training		4 weeks	2/week	↑ balance, ↓ MSWS-12, ↓ postural sway, ↑ step length, ↑ stride length	None
Hogan et al. [26]	66	Group physio-therapy	3–4 mobility section of (GNDS)	10 weeks	1/week	↑6MWT, ↑balance^∗∗^, ↑QOL^∗^, ↓MSIS − 29v2 physical component^∗^, ↓mFIS^∗^	↑balance^∗∗^, ↓mFIS^∗^
	45	1 : 1 physiotherapy		10 weeks	1/week	↑balance^∗∗^, ↑QOL^∗^, ↓MSIS − 29v2 physical component^∗^, ↓MSIS − 29v2 psychological component^∗^, ↓mFIS^∗^, ↑6MWT^∗^	↑balance ^∗∗^, ↓mFIS^∗^, ↑6MWT^∗^
	16	Yoga		10 weeks	1/week	↑balance^∗∗^, ↑QOL, ↓MSIS − 29v2 physical component, ↓MSIS − 29v2 psychological component, ↓mFIS, ↑6MWT	↑balance ^∗∗^
Jackson et al. [21]	4	Kickboxing	6.0-6.5	5 weeks	3/week	↑ balance, gait speed, ↑ Mini-BESTest, ↓ TUG	None
Miller et al. [19]	15	Home ex	6.5–8	8 weeks	2/week	↓ 10WMT, ↓ FIM, ↓ EDSS, ↑ MSIS 29, ↑ muscle strength, ↓ timed sit-stand	None
Giesser et al. [13]	4	BWST	7.0-8.0	20 week	2/week	↓ 10WMT, ↑ 6MWT, ↑ balance, ↑ muscle strength, ↑ QoL, ↓ spasticity (not all patients were able to complete the 10WMT, 6MWT)	None

2MWT: 2 minutes walking test; 5TST: 5-time sit to stand; 6 MWT: 6 min walk test; 10WMT: Ten-Meter Walking Test; ADSTEP: Assistive Device Selection Training and Education Program; BBS: Berg Balance Scale; BFR-W: Blood Flow-Restricted Slow Walking; BWST: Body weight supported training; DGI: dynamic gait index; DST: double support time; EBI: Extended Barthel Index; EDSS: Expanded Disability Status Scale; FAC: Functional Ambulation Category; FSS: Fatigue Severity score; FES: functional electrical stimulation; FSST: Four Square Step Test; FIM: Functional Independence Measure; GNDS: The Guy's Neurological Disability Scale; MAS: Modified Ashworth Scale; mBI: modified Barthel Index; mFIS: modified Fatigue Impact Scale; Mini-BESTest: mini Balance Evaluation System Test: MSIS-29: multiple sclerosis impact scale; MSWS: Multiple Sclerosis Walking Scale questionnaire; ODFS: Odstock dropped foot stimulator; PHQ: patient health questionnaire; QoL: quality of life; RAGT: Robot-Assisted Gait Training; RAGT-VR: Robot-Assisted Gait Training combined with Virtual Reality; rmVO2: resting muscle oxygen consumption; RMI: Rivermead Mobility Index; ROM: Range of motion; SLR: Step Length ratio; TBRST: Total-Body Recumbent Stepper Training; T25FW: timed 25-foot walk test; TST: timed stair test; TUG: Timed Up and Go; VAS: Visual Analogue Scale.; VO2peak: peak oxygen consumption; NR: not reported. Abbreviations are presented in an alphabetical order. ^∗^Statistically significant at *p* ≤ 0.05 or ^∗∗^*p* ≤ 0.001.

## Data Availability

Search results can be obtained by reasonable request of the corresponding author.
